# IoT Framework for a Decision-Making System of Obesity and Overweight Extrapolation among Children, Youths, and Adults

**DOI:** 10.3390/life12091414

**Published:** 2022-09-10

**Authors:** Saeed Ali Alsareii, Ahmad Shaf, Tariq Ali, Maryam Zafar, Abdulrahman Manaa Alamri, Mansour Yousef AlAsmari, Muhammad Irfan, Muhammad Awais

**Affiliations:** 1 Department of Surgery, College of Medicine, Najran University Saudi Arabia, Najran 11001, Saudi Arabia; 2 Department of Computer Science, COMSATS University Islamabad, Sahiwal Campus, Sahiwal 57000, Pakistan; 3 Electrical Engineering Department, College of Engineering, Najran University Saudi Arabia, Najran 11001, Saudi Arabia; 4 Department of Computer Science, Edge Hill University, St Helens Rd, Ormskirk L39 4QP, UK

**Keywords:** IoT, pandemic, obesity, classification, regression, real-time system

## Abstract

Approximately 30% of the global population is suffering from obesity and being overweight, which is approximately 2.1 billion people worldwide. The ratio is expected to surpass 40% by 2030 if the current balance continues to grow. The global pandemic due to COVID-19 will also impact the predicted obesity rates. It will cause a significant increase in morbidity and mortality worldwide. Multiple chronic diseases are associated with obesity and several threat elements are associated with obesity. Various challenges are involved in the understanding of risk factors and the ratio of obesity. Therefore, diagnosing obesity in its initial stages might significantly increase the patient’s chances of effective treatment. The Internet of Things (IoT) has attained an evolving stage in the development of the contemporary environment of healthcare thanks to advancements in information and communication technologies. Therefore, in this paper, we thoroughly investigated machine learning techniques for making an IoT-enabled system. In the first phase, the proposed system analyzed the performances of random forest (RF), K-nearest neighbor (KNN), support vector machine (SVM), decision tree (DT), logistic regression (LR), and naïve Bayes (NB) algorithms on the obesity dataset. The second phase, on the other hand, introduced an IoT-based framework that adopts a multi-user request system by uploading the data to the cloud for the early diagnosis of obesity. The IoT framework makes the system available to anyone (and everywhere) for precise obesity categorization. This research will help the reader understand the relationships among risk factors with weight changes and their visualizations. Furthermore, it also focuses on how existing datasets can help one study the obesity nature and which classification and regression models perform well in correspondence to others.

## 1. Introduction

Obesity refers to excessive amounts of body fat. Obesity is not only caused by food genetics, the environment could also be a cause. The intake of energy and not consuming this energy through physical activity could also be a primary reason for obesity [1]. Obesity is the relationship between calorie intake and energy expenditure. It is a significant health issue associated with chronic illness and has a negative impact and long-term effects on patients and their families. As obesity is a risk factor for a number of diseases worldwide, it can be a threat to the world in the future. The Asia region is already dealing with malnutrition (as many cases have reported). Therefore, the number of obesity cases is increasing significantly with time [2].

Since 1975, the global obesity rate has increased thrice according to the World Health Organization (WHO) [3]. In 2013, the Indonesian Basic Health Research national survey (RISEKDAS) noted that obesity cases were rapidly increasing in Indonesia. Obesity can affect both men and women. The rate of obesity in adult men was 13.9%, 7.8%, and 19.7% in 2007, 2010, and 2013, respectively. In contrast, the rate of obesity in adult women was 14.8%, 15.5%, and 32.9% in 2007, 2010, and 2013, respectively [4]. However, in 2018, according to RISEKDAS (the same survey), the rates decreased to 14.5% in men and 29.3% in women [5].

The 2016 data show that the obesity rate has hit over 650 million people globally [6]. From age 18 and older, the ratio of people who are overweight increased to 39% [7]. Obesity and being overweight lead to other dangerous consequences that could lead to health anxiety. Obesity is the prime reason for significant lifestyle diseases, such as cancer, type II diabetes, lung disease, chronic pulmonary disease, and asthma.

Underdeveloped countries and populations are high victims of these diseases. NCDs (non-communicable diseases) and lifestyle diseases caused 36 million (63%) global deaths in 2008. Of these 36 million people, 80% were affected in underdeveloped countries and the middle class, 13% affected the upper class, and 29% affected those under age 60. Selected literature studies showed an annual increase of 10 million deaths due to NCDs. A survey from 2016 showed an increase of 71% (56.9 million), predicting 75% to 88.5% of deaths (until 2030) from NCDs in emerging countries, while the ratio predicted in developing countries is 65% [8]. Body mass index (BMI) is a primary risk element for the rise in diseases linked to sedentary lifestyles [9]. BMI helps in assessing body composition by calculating “weight/height".² However, BMI is considered a lousy sign of the proportion of body fat because BMI is dependent on age and does not count the fat on different body sites. According to the Institute of Medicine’s 2012 report, there are population-based obesity prevention initiatives that address obesity and being overweight, such as a balanced diet, regular exercise, context- and setting-specific advice, and sound social norms [10].

There are several risk factors associated with obesity. In general, these factors are divided into categories, such as lifestyle factors (e.g., consuming junk food, alcohol, stress, and low physical activity), as well as demographic and socioeconomic elements (e.g., age, gender, marital status, place of residence, and genetic elements) [11]. Some risk factors can be avoided while others cannot. To implement an effective risk reduction strategy, the individual and population levels need to understand the factors that can be avoided [12]. The available data have helped numerous studies in exploring better approaches.

Epidemiological data modeling techniques (using machine learning) are popular in scholarly publications. These techniques can contribute to a better understanding of illness distribution, general health, risk identification, and health risk factors. There are several methods and algorithms available for this purpose [13]. The techniques require exact data classifications to assist in identifying risk detection from the information to lessen the danger signs and morbidity and mortality caused by obesity. Based on data showing compliance with dietary guidelines for obesity prevention, machine learning is applied to predict the likelihood of obesity [14]. Electronic health records are used in machine learning for predicting obesity in children, predicting obesogenic environments for children, and aggregating clinical data, such as metabolomic lipidomics and model drug dose responses [15].

In an online study conducted in Bangladesh (November 2020), 338 adults were examined. Sociodemographic statistics, health-related information, physical activity-related details, and nutrition measurements were all covered in the questionnaire. With two scenarios (’before’ and ’during’ the pandemic commencement) taken into consideration, inferential statistics (i.e., chi-square test, McNemar test) were employed to analyze the relationships between BMI and examined variables [16]. P0.05 was regarded as statistically significant. Results revealed that 30.5% of people were overweight “before” the COVID-19 pandemic and 34.9% of people were overweight “during” the pandemic. This suggests that 4.4% of the participants experienced significant weight gain after the pandemic started.

**Figure 1:**
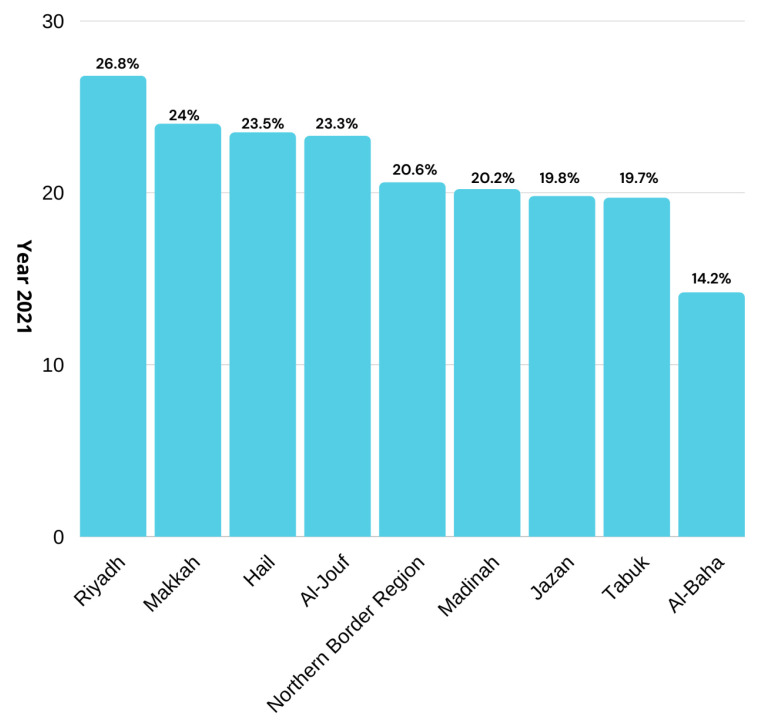
Obesity rate in the provinces of the Saudi Kingdom.

**Figure 2:**
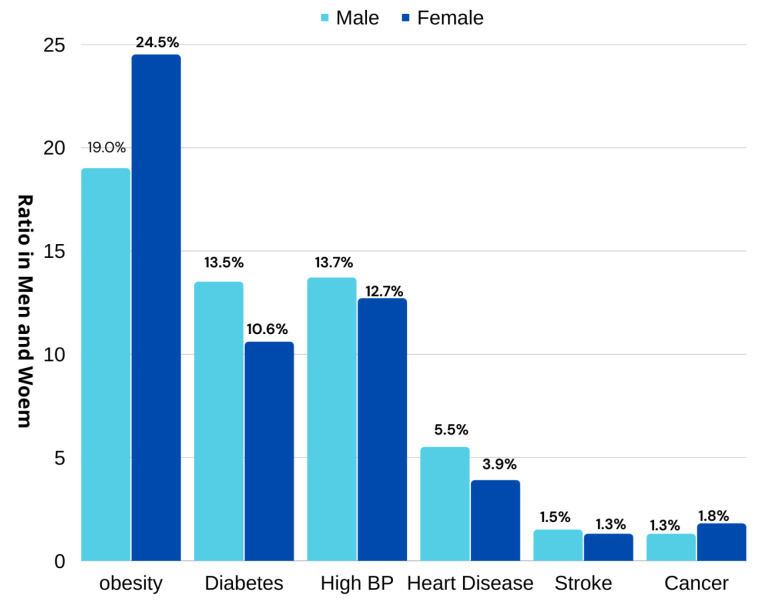
Increased specific disease rate in males and females of the Saudi Kingdom.

A recent report from Riyadh shows that 24.5% of women and 19% of men are suffering from obesity [17]. In 2021, the United Kingdom of Saudi Arabia showed significant increases in obesity rates. The ration varied in men and women but overall statistics showed that there were increases of 26.8%, 24%, 23.5%, 23.3%, 20.6%, 20.2%, 19.8%, 19.7%, and 14.2% in Riyadh, Makkah region, Hail (23.5 %), Al-Jouf, the northern border region, Madinah, Jazan, Tabuk, and Al-Baha, respectively, as depicted in Figure 1. This increase in people being overweight has led to an increase in diseases. The report further revealed that diseases such as obesity, diabetes, high BP, heart illness, stroke, and cancer are rising at ratios of (19.0%, 24.5%), (13.5%, 10.6%), (13.7%, 12.7%), (5.5%, 3.9%), (1.5%, 1.3%), and (1.3%, 1.8%) in both males and females, respectively, as shown in Figure 2.

Several machine learning algorithms are applied with several features to predict specific health conditions. A branch of machine learning known as ANN (artificial neural networks) correlates input parameters and corresponds to output data. ANN has reported several applications in engineering and medicine with variable success rates. Dugan et al. [18] employed artificial intelligence to predict childhood obesity. Six models were used in this research for the study. These models were naïve Bayes, random tree, ID3, j48, random forest, and Bayes net-trained. These models were applied to the clinical decision support system on CHICA. The results showed that ID3 performed well, giving a high ratio of accurate results at 85% and a sensitivity rate of 90%. Jindal et al. used techniques for collective machine learning for obesity prediction. The prediction accuracy proposed for ensemble machine learning approaches was 89.68%. The generalized linear model, partial least squares, and random forest were used in the ensemble prediction through a Python interface.

Hammond et al. [15] used public records and electronic health records for the prediction of obesity in childhood. Several machine learning algorithms were trained for regression and binary classification. The results showed considerable accuracy in the first two years of data collection. The results showed that children at age five could become obese. To distinguish between low, medium, and high obesity, they used logistic regression (using a separate random forest classifier). They employed LASSO regression to ’prophesy’ their continuous BMI values. The bootstrap was run 100 times to obtain a better performance of the model.

Obesity at the national level was predicted using data on food sales; see Dunstan et al. [19]. Three machine learning models were applied to the data obtained from seventy-nine countries. The authors researched basic information from the synergic nature of categories by analyzing food sales. They used five categories. The research considered 60% of countries for 10% (concerning the prevalence range). Moreover, 87% of countries projected the prevalence of obesity with an absolute error of less than 20%. The research showed that baked goods and flour were the most appropriate food categories for the prediction of obesity. Extreme gradient boosting, RF, and SVM were utilized for this model.

Singh and Tawfik [20] presented a machine learning model that might predict adolescent weight gain and obesity. In this study, seven machine learning methods were employed. J48 pruned tree, K-NN, bagging, and other algorithms were used, such as multi-layer perception and random forest. An unaltered and unbalanced dataset was used to vote on the effectiveness of all of the proposed algorithms. The MLP algorithm resulted in a 96% precision value. While the F1-score gave results of 93.96%. Gerl et al. [21] exhibited the use of large population cohorts for the prediction of different measures of obesity. A perplexing lipidomic signature was identified for BFP. A total of 73% of BFP variants were predicted based on age, gender, and lipidome, with the complete range of BFP having mistakes.

Montanezet al. [22] used publicly available genetic profiles and studied machine learning algorithms for predicting obesity. Many machine learning models were involved in this study, such as the SVM algorithm, decision tree, K-NN algorithm, and the decision rule for predicting susceptibility to chronic hepatitis with the help of SNP data. Of all the techniques, SVM produced the best results for the prediction model. According to the simulation findings, the SVM area was below the curve value of 90.5%.

Borrel and Samuel [23] worked on risk mortality and the US adult body mass index category. The effects of obesity and excess weight on the Cox proportional hazard regression were looked at to obtain the death prevalence. They calculated the rate of progress through time for all causes and the mortality rate dependent on peers at a normal weight. They also looked into the mortality rate of persons with obesity/were overweight and had cardiovascular disease. Their proposed results showed CVD caused death in obese adults (over 20% compared to normal-weight adults).

During the pandemic, the obesity rate increased due to lockdowns, and it become extremely important to have digital methods to monitor physical activities and the obeseness of people. Various challenges were involved in the understanding of risk factors and the obesity ratio. Traditionally, statistical analyses were used for understanding obesity, imposing independent linearity and a limited number of prediction sets. Therefore, this study focused on the different machine learning models for the risk identification of obesity. It evaluated the effectiveness of machine learning techniques, such as regression and classification on accessible data in order to compile a list of criteria that could be used to diagnose obesity and being overweight. These results helped us to design an IoT-enabled decision system that might be accessible worldwide where internet facilities are available. Thus, the paper provides the following contributions:

- A novel IoT framework was designed that could be accessible from anywhere and any time for the early prediction of obesity, from the given link http://mlobesity.herokuapp.com/ (accessed on: 22 August 2022)
- A decision-making system was developed with the assistance of state-of-the-art machine learning algorithms.
- The proposed expert system involves both classification and regression models for clear visualization of given data.
- This system could help doctors in making early decisions that might significantly increase the prediction of a patient’s current obesity condition

The remainder of the paper is organized as follows: the proposed machine learning algorithms and IoT system architecture are explained in Section 2. In Section 3 and Section 4, the results and discussion are covered; the conclusion and future work are discussed in Section 5.

## 2. Materials and Methods

Classification and regression are supervised machine learning algorithms used for accurate assessments and instructions. For classification and regression, the process includes the following steps: data collection, preprocessing, data visualization, model training, testing, and evaluating. The research discusses the target population, study sample, and at-risk population. The study does not predict any new risk factors. The sample data focus on the population from the ages >20 to <60, excluding pregnancy and genetic factors.

### 2.1. Dataset Explanation

This study analyzes the data on eating habits and health to estimate the prevalence of obesity among persons from Mexico, Peru, and Colombia. The data were categorized using the values of Insufficient weight, normal weight, overweight level I, overweight level II, obesity type I, obesity type II, and obesity type III, thanks to the class variable NObesity (obesity level) assigned to the records. The dataset consisted of 2111 records and 17 attributes. The SMOTE filter and the Weka tool were used to artificially produce 77% of the data, while a website platform collected 23% of the data directly from users. The dataset is categorized into three parts: **Food intake indicators:** FAVC (frequent consumption of high-calorie foods), FCVC (frequent consumption of vegetables), NCP (number of meals), CAEC (intake of food between meals), CH${}_{2}$O (daily water intake), CALC (alcohol intake). **Body attribute**: TUE (time utilizing technological devices), FAF (regular exercise frequency), SCC (calorie-ingestion tracking), MTRANS (utilized for transportation). **Other attributes**: gender, age, height, weight, smoke, and family history.

(1) $$BMI=\frac{Weight}{height^{2}}$$

Dataset attributes were categorized according to the mass body index as shown in Equation (1) for each individual; the results were compared with the data provided by the WHO and the Mexican normativity.

- Underweight Less than 18.5;
- Normal 18.5 to 24.9;
- Overweight 25.0 to 29.9;
- Obesity I 30.0 to 34.9;
- Obesity II 35.0 to 39.9;
- Obesity III higher than 40.

BMI is considered a ’lousy’ sign relating to the proportion of body fat because BMI is dependent on age and does not count the fat on different body sites. Therefore, a detailed analysis of individual eating habits, physical activities, and other attributes is needed to understand obesity in a better way.

### 2.2. Dataset Preprocessing

Categorical and continuous data were separated into two groups. Classification and regression are considered supervised machine learning algorithms used for accuracy assessment and instruction. The selected dataset had noise—some values were small and some had a considerable enough amount of data for the supervised, trained machine learning model. Data samples containing outliers were discarded; the remaining data were filtered with data mining. Data mining involves clustering, classification, feature selection, association, calculation, outlier analysis, and pattern discovery. Incomplete data were removed during the data cleaning process. Similarly, several steps were involved in the data post-processing, such as pattern interpretation, pattern evolution, pattern visualization, and pattern selection. 1. K-fold assists in the accuracy of the ML (machine learning) model after training. 2. Spyder IDE helps establish a Python environment data science application using anaconda distribution.

### 2.3. Decision Tree

A classification model that recursively divides the datasets into sub-parts is known as a decision tree. There are root nodes, internal nodes in the decision tree, and terminal nodes developed by the subdivision of the tree. Each node was derived from a single parent and could have many child nodes. A decision tree helps in the decision-making process. The context of the decision tree decides the probability of sets. The simple structure of the decision tree has nodes and terminal nodes, which is a supervised approach to classification. Nodes represent the dataset’s properties, and their results are displayed by terminal nodes. C4.5 and random forest are examples of algorithms used to implement the decision tree [24].

### 2.4. Random Forest

Several applications rely on decision tree architecture during training, such as regression and classification; random decision forest is also an ensemble learning technique. Random forest utilizes several decision trees (CART) and then gives the most accurate outcome with the combination of these trees. The decision tree algorithm uses the Gini index technique, which measures the probability that a selected element from the set will be erroneously categorized. The total squared possibility for each class is decreased by 1 from the Gini index calculations. This technique increases the predictive power of the system. Removing the bias created by the decision tree model adds to the system. Additionally, using the “random Forest” R package, random forest can naturally order the relevance of variables in regression or classification tasks [25,26].

### 2.5. Support Vector Machine

SVM offers excellent empirical findings and a strong theoretical base. Several agents have used SVM to complete tasks, including digit recognition, object identification in text categorization, and human activity recognition [27,28,29,30,31]. Based on the ’A’ mathematical model for problems involving regression; classification was supplied by the statistical learning systems. A key benefit of SVM involves the availability of trustworthy tools and techniques for solving issues swiftly and efficiently.

### 2.6. K-Nearest Neighbor

One data mining technique is the K-nearest neighbor (KNN) approach used for categorization, which assigns a batch of data based on learning previously labeled or categorized data. The outcomes of newly categorized query instances based on the majority of the proximity to existing categories in KNN fall under the category of supervised learning, including KNN. The following are processes involved in categorizing using the K-nearest neighbor (KNN) algorithm: 1. Establishes the k parameter; 2. Determines the separation between training and test data using the Euclidean distance calculation; 3. Arranges the formed distances; 4. Establishes the distance closest to the sequence K; 5. Matches the proper class; 6. Assigns the class as the data class to be assessed by counting the number of classes from the nearest neighbors [32].

### 2.7. Naïve Bayes

Naïve Bayes data mining techniques help make predictions in many fields and are used by many researchers. The framework for a hybrid strategy that uses naïve Bayes for parameter optimization and genetic algorithms for prediction is presented in this research. According to the naïve Bayes model, parameters with zero values show weaknesses in the results. This problem can be resolved by applying genetic algorithm optimization. The problem ’suggested’ optimizing genetic algorithms for the study. The study was initialized with an analysis of the literature on the subject of child obesity and adequate data mining models for the prediction of childhood obesity. Following the review, 19 attributes were chosen, and the NB approach was used to predict child obesity. A 75% increase in accuracy was seen in the first test to gauge the utility of the proposed approach [33].

### 2.8. Logistical Regression

Using prior observations from a dataset, a statistical analysis technique called logistic regression predicts a binary outcome, such as yes or no. Using a logistic regression model, a dependent data variable is predicted by looking at the correlation between the independent variables that are already present. For instance, logistic regression may be used to foretell a candidate’s outcome in a political election or whether a high school student will be accepted into a particular college. These simple choices between two options allow for binary outcomes. Thirty input variables were gathered from the patient records, including clinical information (gender, age, body mass index, and concurrent disorders), laboratory testing, and histopathologic results of the gallbladder. The identical database was used to produce a logistic regression model, and similar data were compared to the outcome [34].

### 2.9. IoT Enabled System Architecture

**Figure 3:**
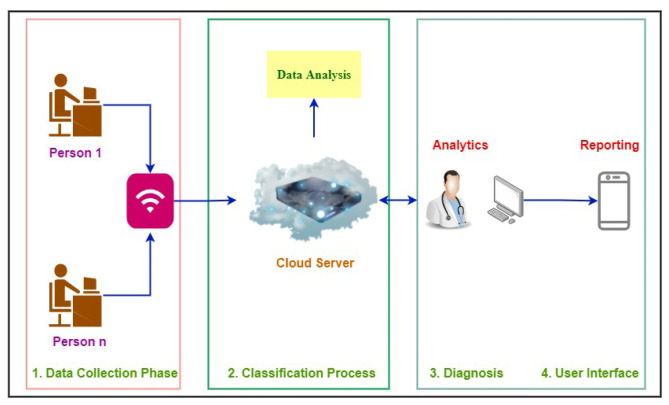
Proposed IoT system architecture.

This system is regarded as a multiple-user access system, allowing numerous users to connect to the cloud simultaneously, as shown in Figure 3. There is only a single universal receiver shared by all users. An IoT system with cloud administration was created to classify obesity. Because it is a distributed system, the cloud is the best solution for a healthcare system that enables doctors to obtain data more easily. Our suggested IoT system comprises four key phases: (1) data collecting, (2) textual data classification, (3) diagnosis, and (4) user interface. Its goal is to lower disease rates through early detection of obesity.

This figure demonstrates that the user is the origin of the entire process. With the web application interface, users engaging with the server and application interfaces are directly coupled. Therefore, when a user interacts with the web interface, a specific request is sent to the server. Upon receiving a request, the server examines it to determine what is the need of the user (obesity prediction, check his/her history, download report, or doctor’s advice). Then the server will decide where to transmit the user’s request after considering the needs of the user. Therefore, the server looks for an expert system that can handle the user’s request and deliver the results. The server assigns the user’s duties after identifying the optimal expert system. The user’s task is inputted into the expert system as a string because the entire model is reliant on textual information, which is utilized to identify obesity in its early stages. After receiving a string input, the algorithm eliminates any extraneous words that are found during the prediction stage. After eliminating superfluous words, the user-provided data are used by the prediction engine to make predictions. Following the calculation of the outcome, the results are sent to the expert system. The server receives the results that the expert system collects. After obtaining the expert system’s results, the server sends it to the web interface, where the user can access his/her results and move forward in light of the report.

## 3. Results

The following metrics help in evaluating machine learning models for classification and regression. **Regression Metrics**: MBE (mean bias error), RMSE (root mean square error), MABE (mean absolute bias error), and R${}^{2}$ (determination coefficients). **Classification metrics**: discuss the classification report and confusion matrix. F1-score, recall, accuracy, and precision are included in the classification report and their equations are shown in Equations (2)–(5). Two dimensions, “actual” and “predicted,” are included in the confusion matrix. For each dimension, there are values for true positive (TruePos), true negative (TrueNeg), false positive (FalsePos), and false negative (FalseNeg).

- True positive: The difference between the actual and anticipated classes is 1.
- True negative: The difference between the actual and projected classes is 0.
- False positive: The predicted class is 1, while the actual class is 0.
- False negative: The predicted class is 0, whereas the actual class is 1.

The following class labels were used for regression and classification purposes: ‘Normal_Weight’, ‘Insufficient_Weight’, ‘Overweight_Level_I’, ‘Overweight_Level_II’, ‘Obesity_Type_I’, ‘Obesity_Type_II’, ‘Obesity_Type_III’ with the indexes of ‘0’, ‘1’, ‘2’, ‘3’, ‘4’, ‘5’, and ‘6’, respectively.

The following formulas help in the calculation of classification metrics:

(2) $$Accuracy=\frac{\left(TruePos+TrueNeg\right)}{TruePos+FalsePos+FalseNeg+TrueNeg}$$

(3) $$Precision\left(P\right)=\frac{TruePos}{TruePos+FalsePos}$$

(4) $$Recall\left(R\right)\;or\;Sensitivity\left(S\right)=\frac{TruePos}{TruePos+FalseNeg}$$

(5) $$F1\text{-}score=2\times \frac{Precision\times Recall}{Precision+Recall}$$

The precision determines how closely the real value resembles the measured value, while accuracy assesses how closely the measured value resembles the actual value. Recall and sensitivity indicate a machine learning model’s overall usefulness. MBE, RMSE, MABE, and R${}^{2}$ are used for regression problems as represented in Equations (6)–(9). If the MBE is low and close to zero, the prediction model performs well. Furthermore, zero represents the optimal situation. The prediction model effectiveness (in the short term) is assessed by the RMSE metric. It always has a positive value, which ought to be close to zero. MABE evaluates the severity of an association. The objective is to come as close to zero. The R${}^{2}$ approach shows how well a method can forecast a set of quantifiable facts. Its value is a number between 0 and 1.

(6) $$MBE=\frac{1}{q}\sum_{n=1}^{q}{\left(b_{n}-c_{n}\right)}^{2}$$

(7) $$RMSE=\sqrt{\frac{1}{q}\sum_{n=1}^{q}{\left(b_{n}-c_{n}\right)}^{2}}$$

(8) $$MABE=\frac{1}{q}\sum_{n=1}^{q}\left|b_{n}-c_{n}\right|$$

(9) $$R^{2}=1-\frac{\sum {\left(b_{n}-c_{n}\right)}^{2}}{\sum {\left(b_{n}-\bar{b_{n}}\right)}^{2}}$$

### 3.1. Confusion Matrix

**Figure 4:**
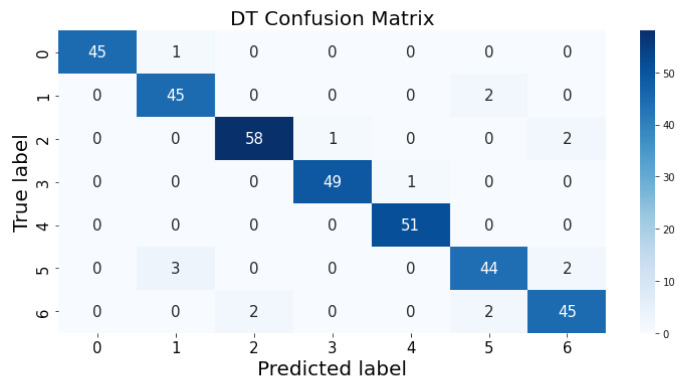
Decision tree prediction on each class testing sample.

**Figure 5:**
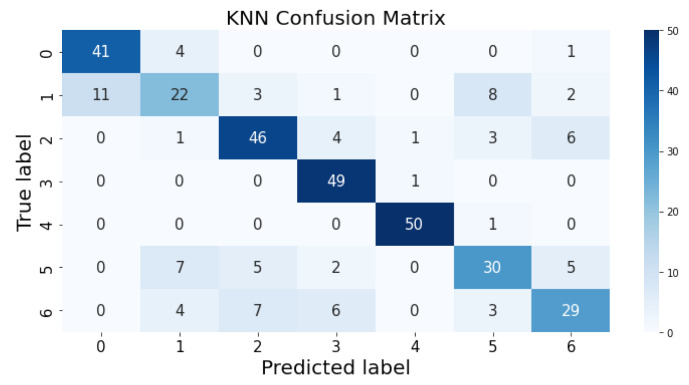
KNN prediction on each class testing sample.

**Figure 6:**
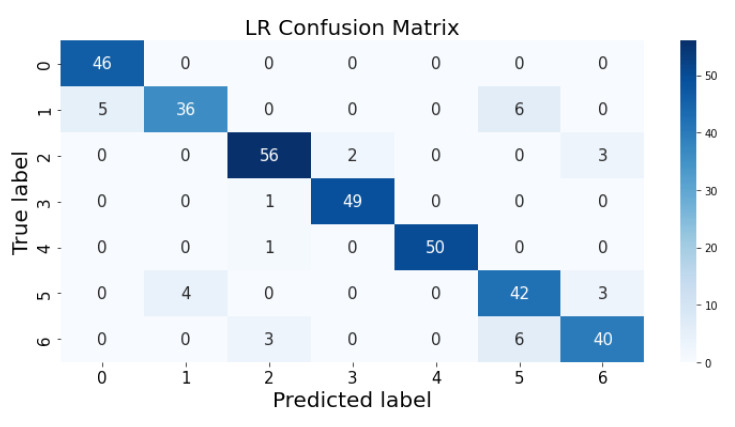
Logistic regression prediction on each class testing sample.

**Figure 7:**
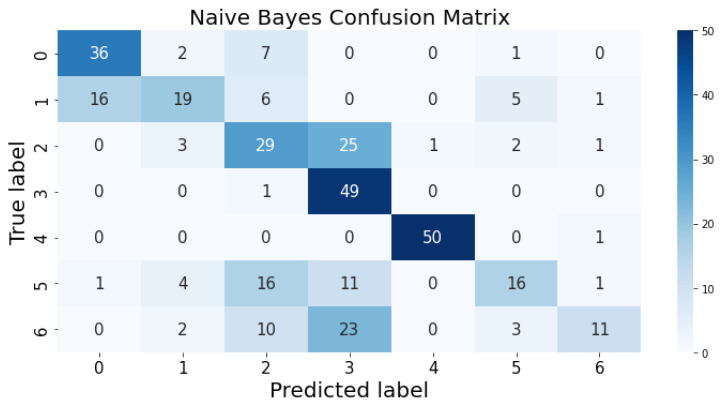
Naïve Bayes prediction on each class testing sample.

**Figure 8:**
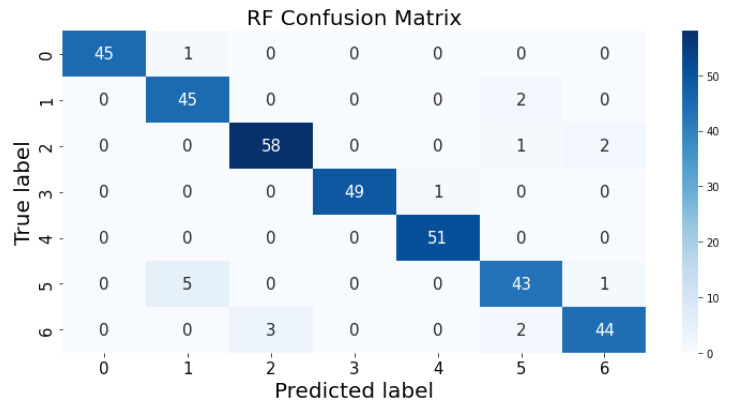
Random Forest prediction on each class testing sample.

**Figure 9:**
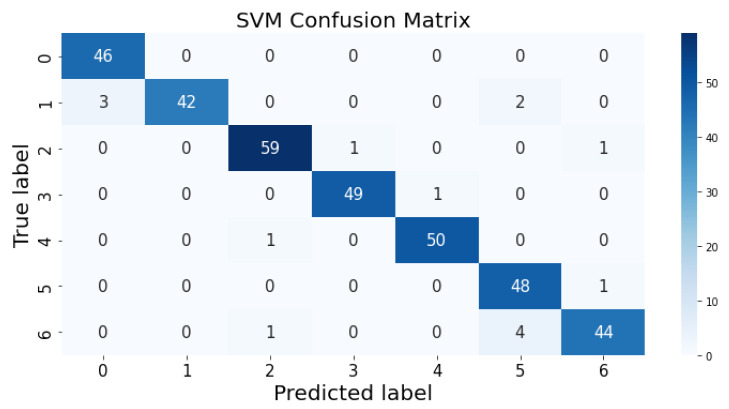
Support vector machine prediction on each class testing sample.

The confusion matrix clarifies the performance of the classification algorithm. The accuracy value can be misled if the number of classes in a dataset is more than one or the dataset has unequal observations. A confusion matrix gives a clear idea of the results of the classification model and highlights the errors. It contains the summary of the predicted results applied to a classified problem [35]. The percentage of accurate classification in all of the predictions is indicated by accuracy. The matrix contains several values, but the confusion matrix tells precisely where the process went wrong. There are two axes in the confusion matrix. The Y-axis shows the test values of the dataset, while the x-axis represents the prediction results of the test values. There are seven classes in the dataset predicted by machine learning algorithms. The confusion matrix of the decision tree, regression logistic, KNN, naïve Bayes, SVM, and random forest are shown in Figure 4, Figure 5, Figure 6, Figure 7, Figure 8 and Figure 9. The colorful boxes represent the actual scores of the classes, while the values in other boxes show the mistaken values.

### 3.2. Real-Time Analysis

**Figure 10:**
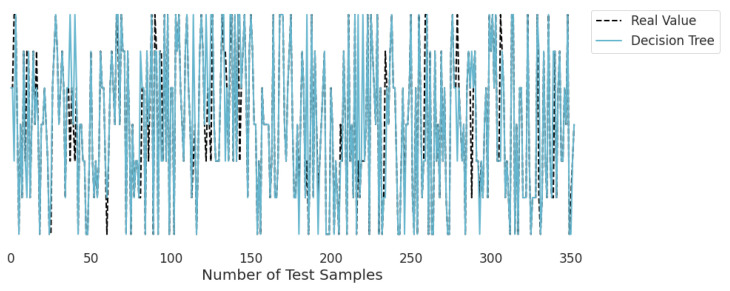
Real-time analysis of each testing sample against the predicted values of the decision tree.

**Figure 11:**
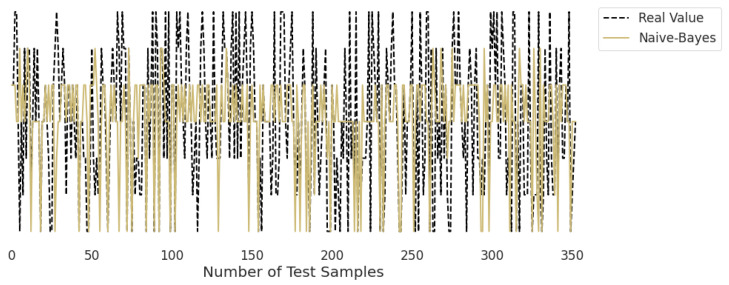
Real-time analysis of each testing sample against the predicted values of naïve Bayes.

**Figure 12:**
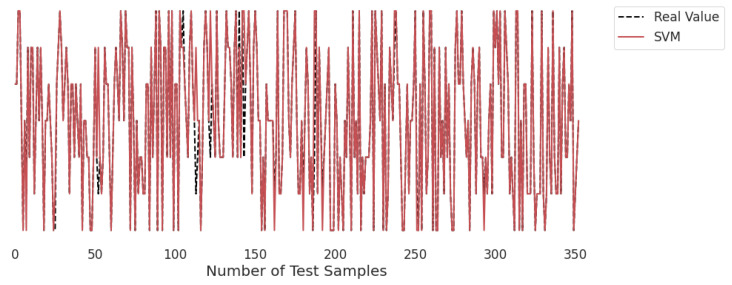
Real-time analysis of each testing sample against the predicted values of SVM.

**Figure 13:**
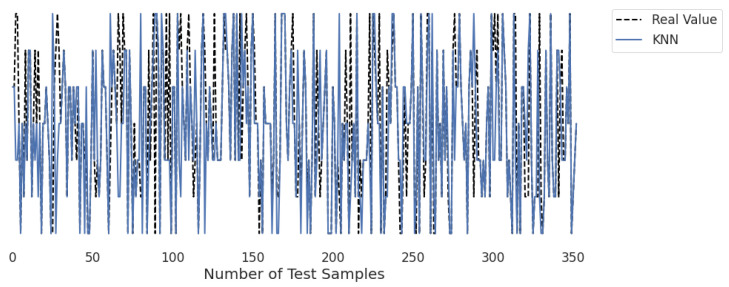
Real-time analysis of each testing sample against the predicted values of KNN.

**Figure 14:**
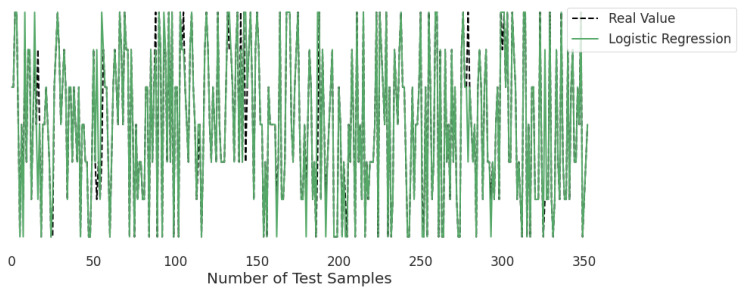
Real-time analysis of each testing sample against the predicted values of logistic regression.

**Figure 15:**
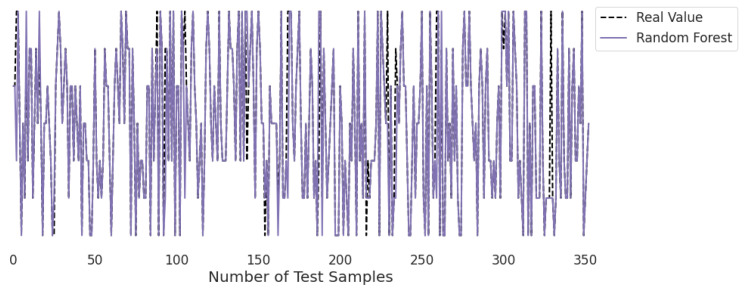
Real-time analysis of each testing sample against the predicted values of random forest.

Figure 10, Figure 11, Figure 12, Figure 13, Figure 14 and Figure 15 represent the predicted and real values of different algorithms. The dotted black line shows the real value that we obtained during the real-time analysis and the colored lines represent the predicted values of the algorithms. These figures map 353 samples of obesity, with a total of 16 columns; each sample value is the sum of 16 columns.

Figure 10 represents the predicted value of the decision tree, which shows that real values match with the predicted values most of the time and provide good results as already described in Table 1 with an accuracy of 95%. This algorithm is able to validate the model by using statistical data, which makes it more reliable.

Figure 11 represents the predicted value of naïve Bayes, which shows that real values did not match with the predicted values most of the time and provided very bad results, as shown in Table 2, with an accuracy of 59%. This algorithm assumes that all predicates are independent and very rarely occur in real life.

Figure 12 presents the predicted value of SVM, which shows that there were very few values where the predicted values did not match the real values and, thus, it provided very good results, as shown in Table 2, with an accuracy of 96%. This algorithm even works with unstructured and semi-structured data.

Figure 13 presents the predicted value of KNN, which shows that there were few values where the predicted values matched the real values and some values where the predicted values did not match the real values; thus, it provided average results, as described in Table 2, with an accuracy of 76%. This algorithm does not perform well on a small dataset.

Figure 14 presents the predicted value of logistic regression, which shows that there were very few values where the predicted values did not match the real values; thus, it provided good results, as shown in Table 1, with an accuracy of 90%. This algorithm is very fast at classifying unknown records.

Figure 15 presents the predicted value of the random forest, which shows that there were very few values where the predicted values did not match the real values and, thus, it provided very good results, as shown in Table 2, with an accuracy of 95%. This algorithm can be used to solve classification and regression problems.

### 3.3. Comparison with Existing Schemes

Table 3 shows a fair comparison between the proposed and existing work. There is a lack of studies on the number of machine learning models and statistical matrices for classification reports. It is not clear how the existing work will perform when the number of algorithms increases.

In [24], two machine learning algorithms—SVM and DT—were used for obesity detection, with predicted precision and recall values, but not the F1-score or accuracy value. Similarly, in [36], there were two machine learning algorithms with prediction values of precision and recall only. In [37], the deep learning approach was adopted for classification purposes. In terms of statistical matrices, only accuracy was considered. These studies do not offer a complete classification report. Furthermore, the existing work does not discuss the error rate in the predicted values. The error rate helps in determining whether the prediction can be considered for further use or not.

However, the proposed system calculates the error rate of the predicted value against each machine learning model. In the proposed work, all machine learning algorithm error rate values were calculated in the form of MBE, RMSE, MABE, and R${}^{2}$. The proposed system utilizes six machine learning models with optimized configuration settings of SVM, KNN, LR, DT, RF, and NB. It also shows the complete analysis of precision, recall, F1 score, and accuracy as shown in Table 4.

## 4. Discussion

This study focused on several machine learning algorithms for early obesity diagnosis. In order to create a list of criteria that could be used to diagnose obesity and being overweight, we assessed the usefulness of machine learning algorithms on accessible data. Our study showed that the SVM performed the best, with the DT and RF classifiers coming in second for early obesity detection. The SVM’s remarkable performance across all experiments may be explained by the fact that it employed an adaptive weighting strategy during training. All selected machine learning models were employed for accuracy, precision, F1-score, and recall. Accuracy evaluates how closely the measured value resembles the actual value; precision measures how closely the real value resembles the measured value. A machine learning model’s recall or sensitivity reveals its usefulness.

The research focused on obtaining maximum outputs from the classifiers by using true positive, false positive, true negative, false negative, the confusion matrix, and classification report, which resulted in precision, F1-score, recall, and accuracy ratios. The metrics listed aid in assessing machine learning models for regression: MABE (mean absolute bias error), RMSE (root mean square error), MBE (mean bias error), and R${}^{2}$ (determination coefficients), whereas classification metrics include the confusion matrix and classification report (F1-score, recall, accuracy, and precision). The dataset for this study included 2111 records. A website platform assisted in collecting 23% of the data directly from users while the SMOTE filter and the Weka tool were utilized to artificially construct 77% of the data.

In terms of MBE, RMSE, MABE, and R${}^{2}$—naïve Bayes predicted the results with a higher error rate and lower determination coefficient value. In terms of MBE, KNN predicted the results with the lowest error rate while the SVM secured the second lowest value. Similarly, the SVM achieved the second lowest value compared to random forest when RMSE, MABE, and R${}^{2}$ results were calculated.

Furthermore, the proposed study utilized maximum machine learning models to obtain a detailed overview of the predicted values as compared to [24,36,37]. The SVM showed the highest value for precision, F1-score, recall, and accuracy, and ’suggested’ the best prediction after taking a close look at the end values. Only accuracy, such as in [37], was not enough to obtain a finer-grained idea of the classification performance. Classifier working was identified by analyzing the value of the precision, F1-score, and recall.

The analysis of seven classes in six machine learning models showed that naïve Bayes had less value for precision, F1-score, and recall. Nonetheless, it is important to point out that the results of this analysis are positive and imply that the suggested SVM can achieve very high performances above 96%.

The proposed IoT system gathers textual data on obesity using data collection tools. The textual data are then communicated to the cloud via the WIFI module, where it goes through preprocessing and classification phases before being scaled to fit the suggested machine learning model, which employs a classifier to detect obesity and extract features from the processed data. The patient can access his/her database to find the classification results during the analytic phase. By submitting the data and receiving the classification report in a couple of seconds, the patient can quickly identify obesity (if there is any). The report is sent to the patient’s doctor in the final step, who will choose the best course of action.

There are certain limitations to the proposed research, despite the fact that it provides considerable potential to address situations in real-life. Due to the lack of accessibility of the datasets gathered from overweight patients, one of these drawbacks is that it exclusively uses datasets that are publicly available. Therefore, in order to categorize the activity patterns, future studies should concentrate on gathering and analyzing the datasets of exclusively fat or overweight persons.

## 5. Conclusions

Obesity is a major public health problem worldwide. The prevalence of obesity has increased dramatically in the past few decades, especially during the COVID-19 pandemic. It is now considered a global epidemic. This is problematic for several reasons, e.g., there is an increased risk of developing serious health conditions, such as heart disease and diabetes. Therefore, we proposed a real-time expert system that successfully determines the possible threat factors related to obesity and being overweight. Several statistical, machine learning, and data visualization methods have been applied to publicly accessible obesity datasets. We performed a fair comparison of machine learning algorithms in terms of precision, recall, F1 score, and accuracy. From the list of proposed algorithms, the SVM outperforms its counterpart schemes. In case of error rates, the following statistical measurements were considered: MBE (MJ/m${}^{2}$), RMSE (MJ/m${}^{2}$), MABE (MJ/m${}^{2}$), and R${}^{2}$. In MBE (MJ/m${}^{2}$), SVM has the lowest error rate nearer to zero, while for RMSE (MJ/m${}^{2}$), MABE (MJ/m${}^{2}$), and R${}^{2}$—random forest has a better performance compared to the others. Our expert system takes input from users via a web interface and passes the data to multiple algorithms to make a classification report. This report will be sent to the patient’s doctor for necessary actions. In this way, we can easily entertain obesity cases in the initial stages.

## Figures and Tables

**Table 1:** Performance analysis of the decision tree, KNN, and logistic regression.

	Decision Tree			KNN			Logistic Regression		
**Classes**	**Precision**	**Recall**	**F1-Score**	**Precision**	**Recall**	**F1-Score**	**Precision**	**Recall**	**F1-Score**
0	1	0.98	0.99	0.79	0.89	0.84	0.9	1	0.95
1	0.92	0.96	0.94	0.58	0.47	0.52	0.9	0.77	0.83
2	0.97	0.95	0.96	0.75	0.75	0.75	0.92	0.92	0.92
3	0.98	0.98	0.98	0.79	0.98	0.87	0.96	0.98	0.97
4	0.98	1	0.99	0.96	0.98	0.97	1	0.98	0.99
5	0.92	0.9	0.91	0.67	0.61	0.64	0.78	0.96	0.82
6	0.92	0.92	0.92	0.67	0.59	0.63	0.87	0.82	0.84
accuracy	0.95			0.76			0.9		

**Table 2:** Performance analysis of random forest, naïve Bayes, and support vector machine.

	Random Forest			Naïve Bayes			Support Vector Machine		
**Classes**	**Precision**	**Recall**	**F1-Score**	**Precision**	**Recall**	**F1-Score**	**Precision**	**Recall**	**F1-Score**
0	1	0.98	0.99	0.68	0.78	0.73	0.94	1	0.97
1	0.88	0.96	0.92	0.63	0.4	0.49	1	0.89	0.94
2	0.95	0.95	0.95	0.42	0.48	0.45	0.97	0.97	0.97
3	1	0.98	0.99	0.45	0.98	0.62	0.98	0.98	0.98
4	0.98	1	0.99	0.98	0.98	0.98	0.98	0.98	0.98
5	0.9	0.88	0.89	0.59	0.33	0.42	0.89	0.98	0.93
6	0.94	0.9	0.92	0.73	0.22	0.34	0.96	0.9	0.93
accuracy	0.95			0.59			0.96		

**Table 3:** Comparison with existing work.

References	Models	Precision	Recall	F1-Score	Accuracy
[24]	SVM	0.62	0.64	-	-
	DT	0.97	0.97	-	-
[36]	NB	0.90	0.91	-	-
	LR	0.90	0.91	-	-
	J48	0.97	0.97	-	-
[37]	DL	-	-	-	0.82
Proposed work	SVM	0.96	0.95	0.95	0.96
	KNN	0.74	0.75	0.74	0.76
	LR	0.90	0.91	0.90	0.90
	DT	0.95	0.95	0.95	0.95
	NB	0.64	0.59	0.57	0.59
	RF	0.95	0.95	0.95	0.95

**Table 4:** Error rates of the predicted values.

	MBE (MJ/m${}^{2}$)	RMSE (MJ/m${}^{2}$)	MABE (MJ/m${}^{2}$)	R${}^{2}$
Decision Tree	−0.006	0.374	0.119	0.901
Naïve-Bayes	0.074	3.62	1.337	−1.057
SVM	0.025	0.235	0.082	0.939
KNN	−0.074	2.125	0.589	0.415
Logistic Regression	0.037	0.643	0.201	0.834
Random Forest	−0.008	0.156	0.054	0.959

## Data Availability

The dataset can be downloaded from the following link: https://archive.ics.uci.edu/ml/datasets/Estimation+of+obesity+levels+based+on+eating+habits+and+physical+condition+ (accessed on 1 June 2022).
